# The impact of socioeconomic status on clinical presentation of multiple myeloma

**DOI:** 10.1016/j.htct.2026.106256

**Published:** 2026-02-11

**Authors:** Lívia Pessôa de Sant'Anna Coelho, Renata Lyrio Rafael Baptista, Gustavo de Almeida Buarque Bretas, Ana Carolina Araujo, Andrea Ribeiro Soares

**Affiliations:** a Hematology Service, Galeão Air Force Hospital, Rio de Janeiro, RJ, Brazil; b Medical Sciences Faculty, Rio de Janeiro State University, Av 28 de setembro, 77, Vila Isabel, Rio de Janeiro, RJ, Brazil; c DOR Institute, Rio de Janeiro, RJ, Brazil; d Hematology Service, Pedro Ernesto University Hospital, Rio de Janeiro State University, Rio de Janeiro, RJ, Brazil

**Keywords:** Multiple myeloma, Socioeconomic status, Clinical presentation, Social class, Education level, Household income per capita, Socioeconomic inequality

## Abstract

**Introduction:**

The influence of socioeconomic status on cancer diagnosis, treatment, and outcomes has been studied for several decades. In multiple myeloma, many authors are investigating the impact of poverty and social inequalities, measured by indicators such as place of residence, number of residents, occupation, income and education, the incidence, stage and management of the disease, and survival, with controversial results. The aim of this study was to ambispectively analyze the association between socioeconomic status and clinical characteristics of multiple myeloma at presentation.

**Methods:**

A total of 296 patients, diagnosed between 2015 and 2023 in three institutions in Rio de Janeiro, Brazil, were included. To assess the socioeconomic status, a social class questionnaire was administered to patients (or relatives, in the cases of death); information about educational level was collected from this interview and medical records and household income per capita were estimated, according to place of residence.

**Results:**

Lower socioeconomic status was associated with delayed diagnosis, symptoms at presentation, advanced stage, poorer performance status, lower hemoglobin and higher calcium values.

**Conclusion:**

These findings suggest a possible relationship between socioeconomic aspects and severity of multiple myeloma presentation in Brazil, and underscore the importance of shaping health policies to promote greater equity in cancer diagnosis and treatment access.

## Introduction

Multiple myeloma (MM) is the second most common hematological malignancy [1]. Its diagnostic criteria were updated by the International Myeloma Working Group (IMWG) and consist of medullary plasmacytosis ≥10 % or plasmacytoma, associated with an end-organ damage (hypercalcemia, renal dysfunction, anemia and/or bone lesion) or, plasmacytosis ≥60 %, ratio of free light chains ≥100 or presence of more than one focal lesion on magnetic resonance imaging (MRI) [2]. Treatment outcomes for MM have evolved significantly in recent decades due to advancements in knowledge of the pathophysiology, earlier detection, and new therapeutic approaches [3, 4, 5]. Novel drugs, especially in the field of immunotherapy, have increased survival rates and improved patients’ quality of life, even in advanced stages [5,6].

However, as in other neoplasms, the effectiveness of new diagnostic and treatment methods does not benefit all MM patients equally [4]. Demographic, social, and economic aspects may influence everything from disease prevention to life expectancy, as they affect exposure to risk factors, the presence and control of comorbidities, access to healthcare, quality of treatment, adherence, and even social and network support [7,8]. In general, it is reported in cancer literature that people with a low socioeconomic status (SES) have more advanced disease at diagnosis, worse general health, [9, 10, 11, 12] less chance of being treated with modern and more efficient drugs and worst outcomes [13, 14, 15, 16]. In MM, the influence of many of these aspects on diagnoses and outcomes has been studied in the last decades, but the conclusions are somewhat controversial [17]. Additionally, most of the studies present some limitations, mainly in the heterogeneity of SES variables, the therapeutic approaches used and outcomes, which can confound analysis [18, 19, 20].

SES can be measured in different ways, based on community or individual characteristics and data from population-based registries or hospital records; the measurement also varies according to the country/region and specific cultural aspects of the population. Common variables used as SES indicators in this kind of research include employment, family or individual income, profession/occupation, level of education, social class, place of residence, ownership of consumer goods, number of people per room/residence, water and sewage networks among others.

Many authors have also been trying to analyze the influence of race/ethnicity on the incidence of MM and outcomes, but it is difficult to know if the observed differences between racial groups are not attributable, at least partially, to socioeconomic inequalities [17,21,22]. In Brazil, assessing the impact of ethnicity is complex, mainly due to an intense population miscegenation and the known prevalence of lower SES among non-white individuals [16,23].

An advanced stage at cancer diagnosis is associated with poorer outcomes, and some studies have shown that this clinical presentation is more severe in individuals with lower SES [13,14]. Forty years ago, when assessing the impact of poverty on the presentation and treatment responses of patients with MM, Savage et al. found that individuals with lower income had more advanced disease at diagnosis and lower overall survival [17]. Since then, many studies that assessed SES aspects in MM populations and confirmed this kind of association, [24,25] but some showed different results [18,26].

The level of education, a commonly used SES indicator, impacts the treatment outcome of MM. In a Chinese study published in 2020 that enrolled 773 MM patients, the authors showed that individuals with less education (secondary school or lower) had a higher likelihood of being elderly, unemployed, rural residents, with a low income and less access to healthcare. This group had more advanced cases (International Staging System [ISS]: III), higher lactate dehydrogenase levels and less frequently received bone marrow transplantation. Furthermore, they had inferior progression-free survival (30.6 versus 58.8 months, respectively) and overall survival (67.5 versus 122.27 months, respectively), when compared with patients with higher educational levels [27].

Therefore, the analysis of the socioeconomic impact on cancer outcomes is of great importance for patients with MM, particularly in countries with significant social inequality, as these aspects may be modifiable risk factors that can affect treatment responses [3,28].

## Objective

This study aimed to evaluate the relationship between SES and clinical characteristics at MM diagnosis of patients treated over the past decade in public and private institutions in Rio de Janeiro, Brazil.

## Methods

Clinical and demographic data were collected retrospectively (January 15- February 21) and prospectively (March 21-February 23) from medical records of all patients diagnosed with MM treated in two public hospitals (one university and one military) and a private institution in Rio de Janeiro, Brazil.

These patients were identified by the diagnosis of MM (ICD10 C90.0) recorded in the patient records and the myelogram registry books of the public hospitals, and by database searches in the computerized system of the private institution.

For each patient, a standardized clinical form was completed containing information on sex, age, date of symptom onset (if available), date of bone marrow (BM) aspiration, performance status (PS), Durie-Salmon (DS) stage, ISS, medullary plasmacytosis, radiologic findings, and laboratory results. Time to diagnosis was defined as the period between symptom onset to the date of diagnostic BM aspiration.

The presence or absence of the following comorbidities at diagnosis was recorded as specified in the clinical records: systemic arterial hypertension, diabetes mellitus, chronic kidney disease, other neoplasms, heart disease, autoimmune disease, psychiatric disorder, thyroid disease, dyslipidemia, chronic obstructive pulmonary disease/asthma, among other conditions. The study was approved by the Research Ethics Committees in March 2021, and all patients or their legal representatives provided written informed consent.

SES was evaluated by three different measures: social class, educational level and household income per capita. To assess social class, the Brazilian Economic Classification Criteria (BECC) was administered to patients, or to a family member, in the case of death [29]. This questionnaire, developed and periodically updated by the Brazilian Association of Research Companies, is used by the Brazilian Institute of Public Opinion and Statistics (IBOPE) in population socioeconomic surveys. The version of the BECC used in this study includes questions about the number of consumer goods (car, washing machine, DVD player, refrigerator, freezer, computer, dishwasher, microwave, motorcycle, and clothes dryer), and the number of domestic employees. It also contains information regarding details of the residence: the number of bathrooms, water supply (distribution network, water well/spring or other means), street characteristics (asphalt or dirt) and educational level of the head of the family (4 years of study or less, 5–8 years, 9 years - full elementary school, complete high/secondary school or complete university). Based on the resulting score (the sum of points obtained in each item), patients were categorized into six social classes: A (45–100 points), B1 (38–44), B2 (29–37), C1 (23–28), C2 (17–22) and DE (0–16). The information about the patient's educational level (when they were not the head of the family) was collected during an interview or from medical records, following the same categories used by the BECC questionnaire. Finally, household income per capita was estimated based on the neighborhood in which patients lived (in Rio de Janeiro city or other cities) using a computer tool designed in 2010 and available for the Brazilian territory. A current value was calculated based on 2010 and 2023 Brazilian minimum wages converted to US dollars [30,31].

To analyze the associations between SES and clinical presentation of MM, patients were categorized as from high (AB) or low (CDE) social classes, high or low educational level (≤9 versus >9 years of study), and with high or low income (according to the median value found). In addition to the main analyses including all patients in the study, the associations between SES and clinical presentation of MM were also specifically evaluated in the subgroup of patients prospectively enrolled.

## Statistical analysis

Descriptive statistics were applied to summarize the data. Associations between social class, educational level, income, and clinical or demographic characteristics at MM diagnosis were assessed using Fisher’s exact test (two-tailed) and the Mann-Whitney U test, for categorical and continuous variables, respectively. All statistical analyses were performed using the Statistical Package for Social Sciences software (version 21.0).

## Results

A total of 296 patients with MM were enrolled in this study (206 retrospectively and 90 prospectively); their main characteristics are shown in Table 1. Of all the patients, 91 % (*n* = 270) had evidence of at least one target organ lesion. Specifically, 60 % (*n* = 160) had anemia, 30 % (*n* = 75) renal dysfunction, 25 % (*n* = 54) hypercalcemia, and 77 % (*n* = 190) osteolytic bone lesions, as determined by radiography, computed tomography (CT), or positron emission tomography (PET) findings. The diagnosis of MM based on new IMWG criteria, such as medullary plasmacytosis ≥60 % or focal lesions on magnetic resonance image (MRI), was made in only 9 % (*n* = 26) of the cases. Comorbidities were present in 75 % of patients and, across all three centers, most individuals had at least one concomitant condition. Systemic arterial hypertension was the most prevalent (53 %), followed by diabetes mellitus (25 %), cardiovascular disease (13 %), thyroid disorders (12 %), and a history of another malignancy (11 %).

**Table 1:** Sociodemographic and clinical characteristics at multiple myeloma diagnosis.

Characteristic	Total
Number of patients - n	296
Institution - n ( %)	
Private	135 (46)
University	111 (37)
Military	50 (17)
Age (median; range)	65.6 (33–93.2)
Female sex - n ( %)	150 (51)
Ethnic group - n ( %)	
White	81 (46)
Non-white	95 (54)
Marital status - n ( %)	
Married	156 (64)
Not married	87 (36)
Performance status ≥2	168 (57)
Comorbidities - n ( %) Systemic arterial hypertension Diabetes mellitus Heart disease Thyroidopathy Other cancer	222 (75) 158 (53) 74 (25) 38 (13) 34 (12) 31 (11)
Classification - n ( %) IgG IgA Light chain Non secretory	172 (64) 57 (21) 35 (13) 6 (2)
Durie Salmon Staging - n ( %)	
I-II	41 (15)
III	230 (85)
International Staging System - n ( %)	
I-II	176 (76)
III	56 (24)
Plasmacytoma - n ( %)	86 (29)
Symptoms at diagnosis - n ( %)	242 (82)
Time to diagnosis in months - *n* = 189 (median; range)	4.7 (0.6–19.1)
Hemoglobin (g/dL) (*n* = 265) (median; range)	9.4 (3.9–16.0)
Creatinine (*n* = 249) (median; range)	1.1 (0.5–10.8)
Calcium (*n* = 190) (mg/dL) (median; range)	9.9 (7.0–17.6)
Albumin - n ( %)	
<3.5 g/dL	94 (45)
≥3.5 g/dL	115 (55)
β2-microglobulin - n ( %)	
<3.5 g/dL	123 (63)
≥3.5 g/dL	73 (37)
Monoclonal protein (*n* = 228) (median; range)	2.7 (0–10)
Medullary plasmacytosis - n ( %)	
<10 %	15 (7)
≥10 %	207 (93)
Place of residence - n ( %)	
Rio de Janeiro	183 (65)
Another city	98 (35)
Status - n ( %)	
Alive	157 (53)
Death	139 (47)
Social class (*n* = 231) - n ( %)	
AB	111 (48)
CDE	120 (52)
Education level - n ( %)	
≥9 years (*n* = 239)	142 (59)
<9 years	97 (41)
Per capita income* (*n* = 280) - n ( %)	
>US$ 397	137 (49)
≤US$ 397	143 (51)

The BECC questionnaire, which collected information on social class, was successfully completed by 78 % of patients or their relatives. Information on educational level was obtained for 81 % of participants, and estimated household income per capita for 95 %. Table 2 summarizes the numbers of patients alive or deceased at the time of socioeconomic data collection and, within each group, the proportion for whom social class, education, or income data were available in each institution.

**Table 2:** Number of patients (alive/deceased) at the time of socioeconomic data collection and availability of information on social class, educational level, and per capita income.

Characteristic	Total (*n* = 296)	University Hospital (*n* = 111)	Military Hospital (*n* = 50)	Private Institution (*n* = 135)
Patients alive at the time of socioeconomic status data collection	157	50	28	79
BECC* respondents (social class)	133 (85 %)	48	27	58
Education level information	134 (85 %)	49	26	59
Per capita income assessment	154 (98 %)	48	27	79
Total with SES^⁎⁎^ information (alive)	156	49	28	79
Patients deceased at the time of socioeconomic status data collection	139	61	22	56
Family members responding to the BECC*	98 (71 %)	44	19	35
Education level information	105 (76 %)	49	19	37
Per capita income assessment	126 (91 %)	56	16	54
Total with SES^⁎⁎^ information (deceased)	135	58	21	56
CCEB Respondents (Social Class)	231 (78 %)	92	46	93
Education Level Information	239 (81 %)	98	45	96
Per Capita Income Assessment	280 (95 %)	104	43	133
Cases with Socioeconomic Information	291	107	49	135

BECC: Brazilian Economic Classification Criteria; SES: Socioeconomic status.

In cases in which at least two of these measures were available, it was observed that individuals from higher social classes had more years of education (p-value <0.001) and higher income (p-value <0.002), and also, those with higher educational level had higher income (p-value <0.001). A total of 291 cases had at least one SES measure and were included in the association analyses.

Patients from higher social classes had a higher frequency of comorbidities (p-value = 0.048), compared to those from lower classes. However, patients from lower social classes had a longer time from first symptom to the date of bone marrow evaluation (p-value = 0.025), a higher proportion of DS stage III (p-value = 0.002) and hemoglobin values ​​<8.5 g/dL (p-value = 0.03), as well as higher calcium values (p-value = 0.019), when compared to patients from higher social classes (Table 3).

**Table 3:** Associations between social classes and clinical presentation of multiple myeloma.

Characteristic	Total	Social classes AB	Social classes CDE	p-value
Comorbidities - n ( %)	173 (75)	90 (81)	83 (69)	0.048
Durie Salmon - n ( %)				
I-II	28 (13)	21 (21)	7 (6)	0.002
III	182 (87)	78 (79)	104 (94)	
Time to diagnosis in months (median; range)	*n* = 151 4.8 (0.6–19.1)	4.0 (0.8–16.0)	5.9 (0.6–19.1)	0.025
Hemoglobin (g/dL) - n ( %)				
<8.5 g/dL	77 (37)	27 (29)	50 (44)	0.030
≥8.5 g/dL	130 (63)	63 (56)	67 (71)	
Calcium (mg/dL) (median; range)	10.0 (7.0–17.6)	9.6 (7.0–16.6)	10.2 (7.5–17.6)	0.019

Of the individuals who studied up to nine years, there was a higher proportion of females (p-value = 0.004), patients with PS ≥2 (p-value = 0.025) and delayed diagnosis (p-value = 0.011) than those with more than nine years of study (Table 4).

**Table 4:** Associations between level of education and clinical presentation of multiple myeloma.

Characteristic	Total	Educational level >9 years	Educational level ≤9 years	p-value
Sex - n ( %)				
Male	126 (53)	86 (61)	40 (41)	0.004
Female	113 (47)	56 (39)	57 (59)	
Performance Status - n ( %)				
0–1	105 (44)	71 (50)	34 (25)	0.025
≥2	134 (56)	71 (50)	63 (65)	
Time to diagnosis in months (median; range)	*n* = 157 4.8 (0.6–19.1)	4.2 (0.6–16.0)	6.2 (0.8–19.1)	0.011

Individuals with higher estimated household incomes per capita were older than those with lower income (p-value = 0.009). Additionally, a higher proportion of patients with lower income presented with PS ≥2 (p-value = 0.011) and with symptoms at diagnosis (p-value = 0.034). Among these individuals, the median hemoglobin value was lower (p-value = 0.044), when compared to those with higher income (Table 5). No other statistically significant associations were found between SES measures and characteristics at diagnosis.

**Table 5:** Associations between income and clinical presentation of multiple myeloma.

	Per capita income			
Characteristic	Total	Per capita income >US$397	Per capita income ≤US$397	p-value
Age (median; range)	65.5 (33.0–93.2)	67.0 (33.0–93.0)	62.0 (35.8–93.2)	0.009
Performance Status - n ( %)				
0–1	125 (45)	72 (53)	53 (37)	0.011
≥2	155 (55)	65 (47)	90 (63)	
Symptoms at diagnosis - n ( %)	227 (81)	104 (76)	123 (86)	0.034
Hemoglobin (g/dL) (median; range)	9.3 (3.9–16.0)	9.8 (4.2–16.0)	9.1 (3.9–15.0)	0.044

In the separate analysis of the 90 patients prospectively included from March 2021 onward, similar associations were observed between SES and characteristics of clinical presentation of MM, including age at diagnosis, presence of comorbidities, time to diagnosis, and serum calcium levels. Additionally, this analysis revealed that lower social class was associated with a higher frequency of plasmacytoma, while lower income was associated with higher calcium levels and lower serum albumin concentrations (Table 6).

**Table 6:** Associations between SES (social classes and income) and clinical presentation of the group of multiple myeloma patients enrolled prospectively.

p-value	Social classes CDE *n* = 40	Social classes AB *n* = 40	Total *n* = 80	Characteristic
0.031	59.9 (33.7–89.7)	67.3 (40.3–87.0)	64.3 (33.7–89.7)	Age (median; range)
0.031	22 (55)	32 (80)	54 (68)	Comorbidities - n ( %)
0.031	18 (45)	8 (20)	26 (33)	Plasmacytoma - n ( %)
0.010	6.8 (0.6–19.1)	3.8 (0.9–8.1)	4.8 (0.6–19.1)	Time to diagnosis in months (median; range)

p-value	Per capita income ≤US$397 *n* = 43	Per capita income >US$397 *n* = 45	Total *n* = 88	Characteristic

0.001	19 (53)	5 (15)	24 (34)	Hypercalcemia (mg/dL) - n ( %)
0.005	11.1 (9.4–17.4)	10.0 (8.4–15.8)	10.4 (8.4–17.4)	Calcium (mg/dL) - median; range
0.034	3.3 (1.8–4.6)	3.7 (2.8–4.8)	3.5 (1.8–4.8)	Albumin (g/dL) - median; range

## Discussion

The role of specific socioeconomic factors on the clinical presentation of MM remains understudied. This research aimed to address this gap by examining the association between social class, educational level, household income per capita, and individual characteristics at diagnosis of 291 patients followed in three onco-hematological centers in Rio de Janeiro, Brazil.

A lower prevalence of concomitant diseases, observed among patients from lower social classes, may indicate that comorbidities are underdiagnosed in these individuals, mainly due to lack of awareness of their early symptoms and to difficulty in accessing health services. Probably, patients from lower social classes seek health services more for therapeutic rather than preventive treatments. In an analysis of the sociodemographic profile of Brazilian health service users, some authors described that 58 % of public services visits were aimed at disease management, while just 29 % focused on preventive measures. On the other hand, in private services, these proportions were, respectively, 44 % and 34 % [32].

In the present study, MM patients from lower social classes were more frequently in advanced DS stage, with lower hemoglobin and higher calcium levels, and it took longer for them to be diagnosed, a situation that also happened with those with a lower educational level. Although there are few organized national data on the prevalence, profile and outcomes of hematological cancer patients, it has been observed that severity at diagnosis is higher than that described in developed nations and, within Brazil, it is even worse among poorer compared to richer patients. In recent publications from the Brazilian Hodgkin's Lymphoma Registry, authors described a higher prevalence of advanced stages and a longer time from the first symptom to diagnosis among patients from less favored social classes; in these studies, researchers also used the BECC questionnaire [8,33]. Similar findings are also described in non-Hodgkin's lymphoma [34] and leukemia populations [35,36].

The association observed between lower educational level and female sex probably reflects this kind of gender inequality in the older Brazilian population. According to national data from 2022, schooling rates in the country for men and women aged between 18 and 24 were 28.1 % and 32.6 %, respectively, However, when age groups similar to the sample studied here were evaluated, slightly higher illiteracy rates are observed among women (16.3 % versus 15.7 %) [37]. Furthermore, patients with less than nine years of education, as those with lower income, presented with a poorer PS (≥2), reflecting more limitations in work and self-care daily activities and restriction to bed for a greater percentage of time during the day when compared to those with higher educational levels and incomes.

The association between lower income and lower median age differs from Brazilian Family Budget Surveys [38]. Data from 2017/18 showed that people aged 65 or older had lower household incomes per capita than those between 25 and 64 years old. Lower income was also associated with symptoms at diagnosis and with lower median hemoglobin levels, likely reflecting the greater clinical severity of this unfavored group.

Acknowledging the limitations inherent to retrospective analyses, a specific evaluation was performed on the subset of prospectively included cases. The findings of this analysis were consistent with the overall cohort, suggesting an association between lower SES and a more severe clinical presentation of MM. Even so, further investigations using rigorous prospective designs are warranted to validate and clarify this relationship.

The results of this study constitute a meaningful advance in the Brazilian context, revealing potential associations between socioeconomic factors and critical clinical variables, including time to diagnosis, DS stage, PS, hemoglobin and calcium levels, and the presence of symptoms at diagnosis. These findings align with the perceptions of healthcare professionals caring for MM patients and are reinforced by real-world evidence from the Brazilian healthcare system.

## Conclusion

The present data concerning social class, educational level, and household income per capita of MM patients and their association with the disease's clinical presentation contribute significantly to enhancing our understanding of MM management in underdeveloped countries. This information can support discussions surrounding healthcare policies aimed at promoting equiTable access to timely MM diagnosis, an essential condition for optimizing therapeutic outcomes, enhancing quality of life, and extending survival rates for affected individuals. Given that the majority of MM patients globally reside in less developed nations, tackling these concerns has the potential to notably enhance their overall health outcomes. Nonetheless, additional evaluations, preferably prospective and involving larger patient cohorts across multiple healthcare institutions, are warranted to validate these initial findings.

## Data availability statement

The data that support the findings of this study are available from the corresponding author upon reasonable request.

## Conflicts of interest

None.
